# GLP-1 Analogs Reduce Hepatocyte Steatosis and Improve Survival by Enhancing the Unfolded Protein Response and Promoting Macroautophagy

**DOI:** 10.1371/journal.pone.0025269

**Published:** 2011-09-21

**Authors:** Shvetank Sharma, Jamie E. Mells, Ping P. Fu, Neeraj K. Saxena, Frank A. Anania

**Affiliations:** Division of Digestive Diseases, Department of Medicine, Emory University School of Medicine, Atlanta, Georgia, United States of America; Istituto Nazionale per le Malattie Infettive, Italy

## Abstract

**Background:**

Nonalcoholic fatty liver disease (NAFLD) is a known outcome of hepatosteatosis. Free fatty acids (FFA) induce the unfolded protein response (UPR) or endoplasmic reticulum (ER) stress that may induce apoptosis. Recent data indicate ER stress to be a major player in the progression of fatty liver to more aggressive lesions. Autophagy on the other hand has been demonstrated to be protective against ER stress- induced cell death. We hypothesized that exendin-4 (GLP-1 analog) treatment of fat loaded hepatocytes can reduce steatosis by autophagy which leads to reduced ER stress-related hepatocyte apoptosis.

**Methodology/Principal Findings:**

Primary human hepatocytes were loaded with saturated, *cis*- and *trans*-unsaturated fatty acids (palmitic, oleic and elaidic acid respectively). Steatosis, induced with all three fatty acids, was significantly resolved after exendin-4 treatment. Exendin-4 sustained levels of GRP78 expression in fat-loaded cells when compared to untreated fat-loaded cells alone. In contrast, CHOP (C/EBP homologous protein); the penultimate protein that leads to ER stress-related cell death was significantly decreased by exendin-4 in hepatocytes loaded with fatty acids. Finally, exendin-4 in fat loaded hepatocytes clearly promoted gene products associated with macroautophagy as measured by enhanced production of both Beclin-1 and LC3B-II, markers for autophagy; and visualized by transmission electron microscopy (TEM). Similar observations were made in mouse liver lysates after mice were fed with high fat high fructose diet and treated with a long acting GLP-1 receptor agonist, liraglutide**.**

**Conclusions/Significance:**

GLP-1 proteins appear to protect hepatocytes from fatty acid-related death by prohibition of a dysfunctional ER stress response; and reduce fatty acid accumulation, by activation of both macro-and chaperone-mediated autophagy. These findings provide a novel role for GLP-1 proteins in halting the progression of more aggressive lesions from underlying steatosis in humans afflicted with NAFLD.

## Introduction

Non-alcoholic fatty liver disease (NAFLD) is a spectrum of chronic diseases including fatty liver, or bland steatosis, as well as more aggressive lesions including steatohepatitis, lobular necroinflammation with fibrosis, or cirrhosis. NAFLD-related cirrhosis can lead to end-stage liver disease and hepatocellular carcinoma (HCC). Like other chronic end-stage liver diseases the major option for afflicted persons is liver transplantation. NAFLD appears to be associated with obesity and insulin resistance, and is considered the hepatic manifestation of the metabolic syndrome, along with type 2 diabetes mellitus (T2DM) and certain dyslipidemias [1]. An important component of Western diets includes both saturated fatty acids as well as trans-saturated fatty acids. Recent evidence indicates that such fatty acids are partly, responsible for inducing steatosis and fueling hepatocyte insulin resistance [2].

GLP-1 is an incretin that is released from the ***L***-cells of the small intestine which targets pancreatic ß-cells to release insulin and reduce glucagon production in response to food intake [3]. Insulin resistance also results in defective glucagon like peptide-1 (GLP-1) release [4]. Recently we demonstrated that GLP-1 receptors are present on human hepatocytes [5] and that exposure of hepatocytes to GLP-1 receptor agonists led to a reduction of fat load in hepatocyte cell lines, HepG2 and Huh7. Little work, however, has been performed in primary human hepatocytes *in vitro* to elucidate the lethal potential of such fatty acids discussed previously [6].

Fatty acids are known to induce the unfolded protein response (UPR), a compensatory cellular mechanism to handle cell stress [7]. This process, including protein degradation and inhibition of translation allows cells to survive stress caused by defective proteins. In addition to the UPR an additional component to maintain a healthy proteome in the cell - lysosomal degradation or autophagy, has been shown to be critical in removing potentially toxic fatty acids from cells. In addition to defective protein degradation, lysosomes have also been shown to degrade other intracellular components, including whole organelles, lipid deposits, proteinaceous inclusions and aggregates [8], [9]. Singh et al. (2009) recently demonstrated that a fatty acid load in mouse hepatocytes is reduced by macroautophagy [10]. Another type of autophagy, chaperone mediated autophagy (CMA) has also been demonstrated to reduce cell stress by removing proteins that have a signal sequence [11]. While the molecular details regarding chaperone mediated autophagy (CMA) are less well-developed regulators of CMA have recently been identified in murine hepatocytes [12].

Investigations exploring the role of GLP-1 in fatty acid-induced pancreatic beta cell UPR have demonstrated that excitation of GLP-1 receptor in ß-cells leads to improvement in cell survivability [13], [14], [15]; however it is not known whether GLP-1 reduced the fatty acid burden by regulating either macroautophagy or lipoautophagy; or whether chaperone mediated autophagy plays a beneficial role. With this background we investigated the ability of human hepatocytes (*in vitro*) and murine hepatocytes, in an *in vivo* animal model of high fat high fructose feeding, to handle hepatocyte steatosis in response to GLP-1 analogs.

## Results

### Primary Human Hepatocytes

#### Exendin-4 reduces steatosis and enhances survivability in hepatocytes

**Figure 1:**
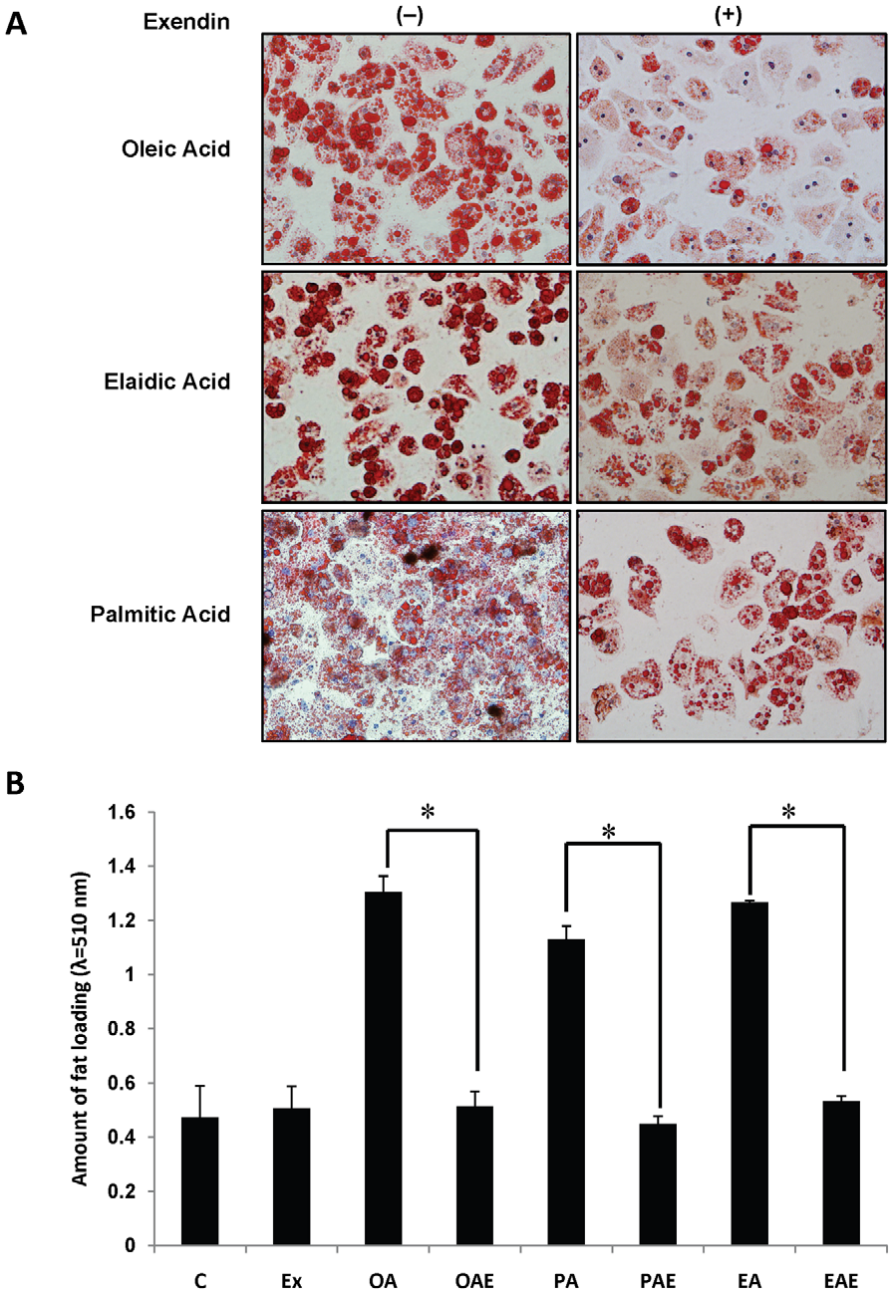
Exendin-4 treatment reduces fatty acid load in hepatocytes. Primary human hepatocytes were treated with fatty acids for 24 h and then supplemented with exendin-4 in fresh media. Hepatocytes treated with fatty acids were cultured for additional 24 h. (**A**) Primary human hepatocytes treated with exendin-4 (**+**) demonstrate reduction of fat load as quantified by Oil Red O staining of intracellular lipid droplets (**B**). (***C*** *:* control; ***Ex***: exendin-4; ***OA***: oleic acid; ***OAE***: oleic acid with exendin-4; **PA**: palmitic acid; ***PAE***: palmitic acid with exendin-4; ***EA***: elaidic acid; ***EAE***: elaidic acid with exendin-4). Three independent experiments with three replicates each were conducted *p<0.001, Tukey-Kramer.

Very limited published studies have investigated models of steatosis in primary human hepatocytes. We employed primary human hepatocytes to confirm their potential as in vitro model to study hepatocyte steatosis. Fat loading was observed by staining the lipid droplets with Oil red O. Microscopic observations of free fatty acids (FFA) treated primary human hepatocytes revealed that conspicuous steatosis (Fig. 1A) were caused by the *cis*-, *trans*-unsaturated, and saturated fatty acids (oleic, elaidic and palmitic respectively). We treated human hepatocytes engorged with FFA with exendin-4 to first determine if it led to reduction in FFA stores; and, whether exendin-4 protected such hepatocytes from apoptosis. We observed that steatosis was significantly reduced after exendin-4 treatment (Fig.1A). Quantification of Oil red O stain corroborated these findings and demonstrated a fifty percent reduction in fat load regardless of the FFA used for fat-loading (Fig. 1B).

**Figure 2:**
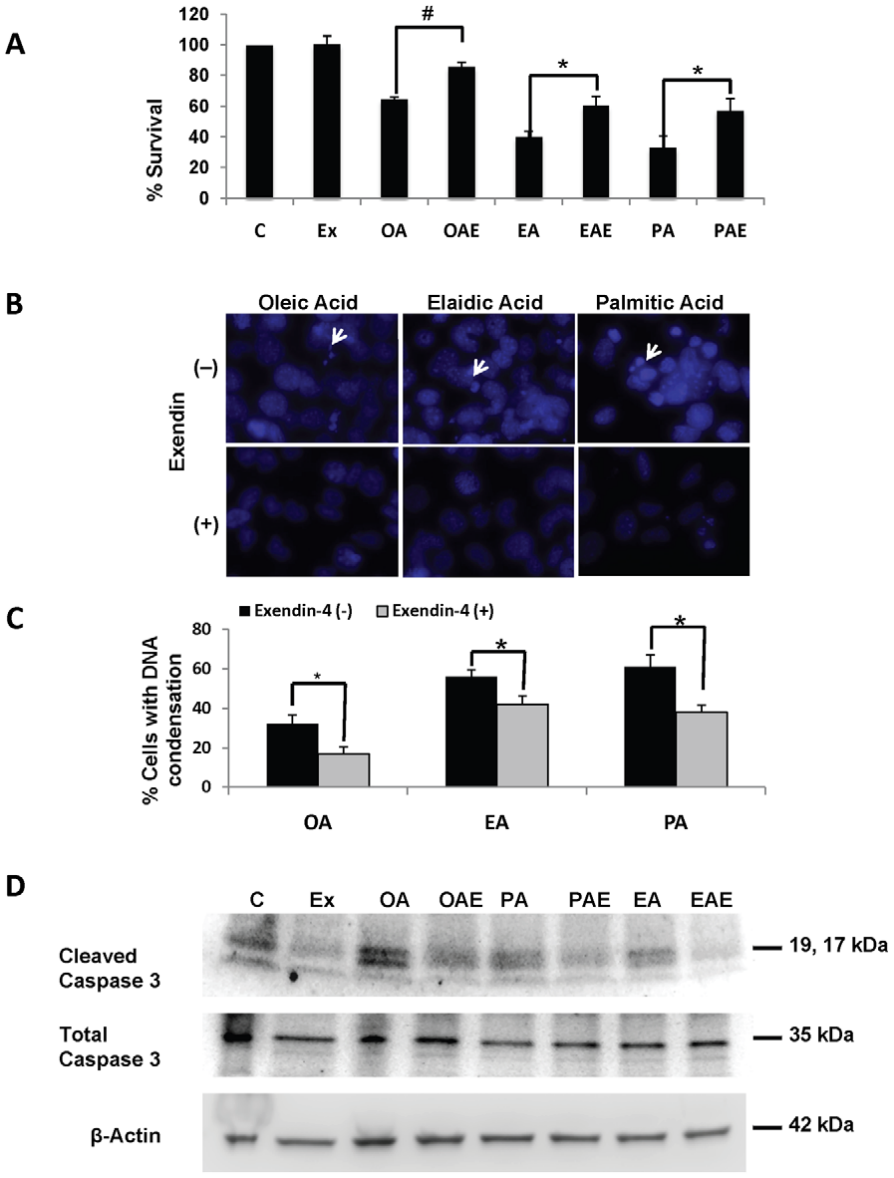
Exendin-4 suppresses apoptosis in primary human hepatocytes. (**A**) Hepatocyte survival increased after exendin-4 treatment as measured by XTT assay. (**B**) DNA condensation as observed by DAPI staining of cells treated with different fatty acids and exendin-4. Damaged DNA is marked by white arrows (**C**). (**D**) Exendin-4 treatment led to suppression of apoptosis as demonstrated by reduction in cleaved caspase 3. These experiments were performed three times in triplicate, #p<0.001, *p<0.05, Tukey-Kramer.

Hepatocytes treated with fatty acids alone demonstrated reduced survival as measured by cleavage of tetrazolium salt XTT to correlate hepatocyte viability. Cells that were treated with exendin-4 showed a higher dye concentration in comparison to cells with FFA alone (Fig. 2A). This suggested that exendin-4 resulted not only in reduction of FFA stores in the cells, but also improved mitochondrial function and survival. On examination of fat loaded and exendin treated cells with DAPI, we found a reduction in DNA condensation in comparison to only fat loaded cells (Fig. 2B). Quantification of the percent cells with DNA condensation (Fig. 2C) confirmed our observations. To confirm if this improvement was a result of reduced apoptosis we examined cleaved caspase 3 levels in cells treated with exendin-4. Immunoblots revealed that there was a marked decrease in cleaved caspase 3 in exendin treated cells (Fig. 2D), supporting our survivability assay.

#### Fatty acid induced ER stress is mitigated by GLP-1 analogs

**Figure 3:**
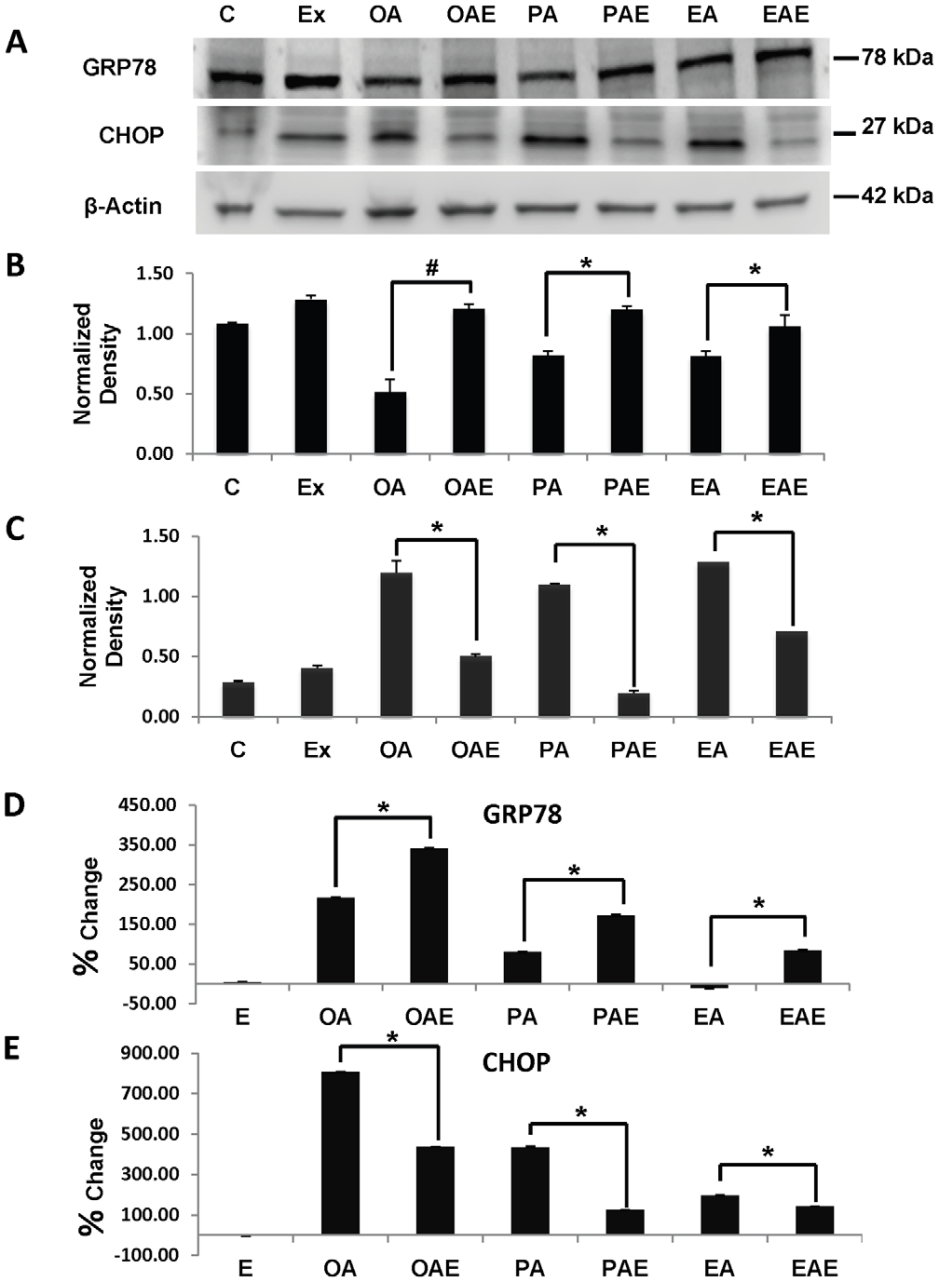
GLP-1 analogs reduce ER stress in hepatocytes. (**A**). Immunoblot of ER stress marker proteins GRP78 and CHOP. (**B**) GRP78 protein levels were increased in following primary hepatocyte cultures in presence of exendin-4 for 24 h. GRP78 levels were significantly increased by oleic acid more than by either palmitic or elaidic acid. (**C**) CHOP expression was significantly reduced by exendin-4, with maximum reduction from lysates obtained from elaidic acid-loaded hepatocytes. (**D**–**E**) Percent change over control in the expression levels of GRP78 and CHOP in human hepatocytes as calculated by RT-qPCR analysis. Representative blots from different independent experiments are presented here. #p<0.001, *p<0.05, Tukey-Kramer.

It is well known that FFA causes ER stress [7], [16], which if uncontrolled can lead to cell death by apoptosis. Apoptotic cell death is believed to be the principle mechanism whereby NAFLD can progress to more significant lesions including non-alcoholic steatohepatitis (NASH) and NASH-related hepatic fibrosis [17]. Phosphorylation of PERK and splicing of XBP were used as markers to confirm setting in of ER stress (Fig. S1- A, C). PERK phosphorylation was higher in fat treated cells but did not show difference after exendin-4 treatment from the fat loaded cells (Fig. S1- A, B). XBP splicing indicated ER stress establishment in fat-only treatments, which was suppressed after exendin-4 (Fig. S1- C: OA, PA, and EA). From the data we concluded that hepatocytes were under ER stress after fat treatments. We speculated that cell death observed in primary hepatocytes may be a consequence of the inability of the ER stress mechanism to handle excess unfolded proteins resulting from excess stress due to FFA-hepatocyte loading. To verify our concerns, we measured the expression of glucose regulated protein, GRP78, the key chaperone protein that mediates the ER stress response to prevent its dysfunction and ultimately prevents cell death. Immunoblots demonstrated that exendin-4 treatment of elaidic acid-loaded hepatocytes (EAE) led to a significant increase in GRP78 levels in contrast to levels determined in elaidic acid loaded hepatocytes (EA) alone (Fig. 3A, B). Exendin-4 significantly increased GRP78 mRNA expression in all FFA-loaded cells when compared to FFA-loaded hepatocytes alone (Fig. 3D). Interestingly, oleic and palmitic acid led to an increase of GRP78 at the message level but not at the protein level, which was lower than the control.

C/EBP homologous protein (CHOP) has been identified as a crucial link between a dysfunctional ER stress and apoptosis and is a highly expressed gene during ER stress [18]. Corresponding to the increased ER stress caused by FFAs, CHOP protein levels were increased. In contrast, exendin-4 reduced CHOP expression both at the protein and mRNA levels regardless of the FFA type loaded (Fig. 3A, C, and E). It is noteworthy that while all the fatty acids induced the expression of CHOP, exendin-4 treatment suppressed the levels three fold in both oleic and palmitic acid loaded hepatocytes in comparison to elaidic acid. We did not find significant difference in 18s rRNA expression levels between treatments (Fig. S2).

#### GLP-1 reduces fat load in hepatocytes by inducing autophagy

**Figure 4:**
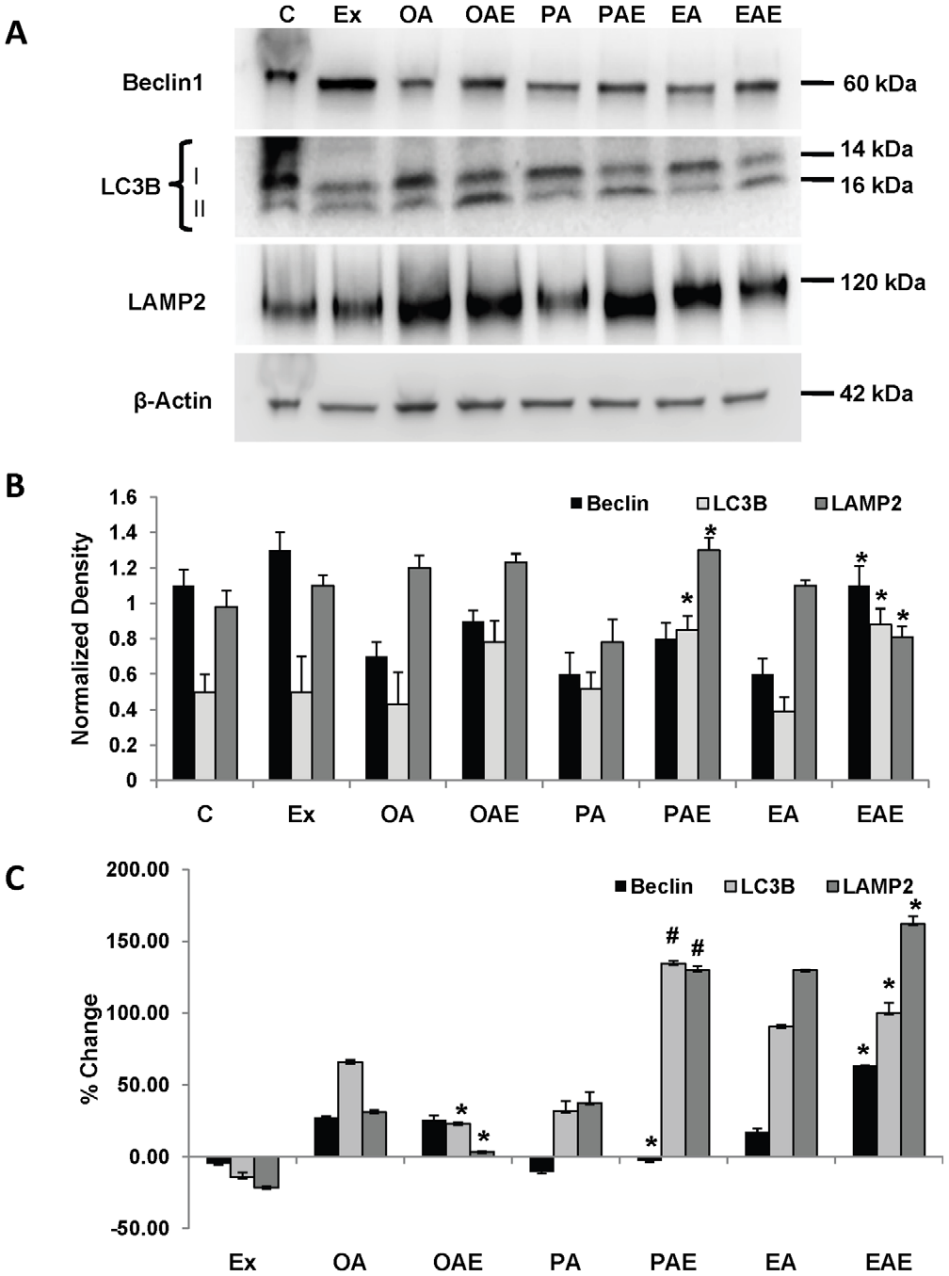
GLP-1 analogs induce macroautophagy and lysosomal turnover. (**A**) Immunoblot of human hepatocytes for autophagy related proteins – beclin-1, LC3B and LAMP2A. Note the increased conversion of LC3B-I to LC3B-II in samples treated with exendin-4. (**B**) Densitometric analysis of immunoblots. For LC3B 16 kDa band was quantified. (**C**) Beclin, LC3B and LAMP2A gene expression levels were elevated in exendin-4 treated cells, as quantified by RT-qPCR. In (**B**) and (**C**) significance was determined by comparing exendin-4 treated samples vs. its respective fat loaded sample for each gene. These experiments were repeated three times in triplicate, #p<0.001, *p<0.05, Students t test.

Recently, macroautophagy has been identified as a mechanism for removal of fatty acid loads from hepatocytes. We wanted to confirm if GLP-1 analogs induced autophagy in fat loaded cells. Examination of autophagy related genes and proteins revealed that GLP-1 analog treatments both *in vitro* and *in vivo* increased key proteins associated with macroautophagy. Beclin and LC3B-II are two key proteins associated with macroautophagy. Exendin-4 significantly increased beclin protein levels except in oleic acid-treated hepatocytes where the change was insignificant; and exendin-4 also significantly increased the conversion of LC3-B1 to LC3B-II (Fig. 4A, B).

These observations were in agreement with mRNA expression levels also. Beclin expression levels were decreased in response to saturated fatty acid in contrast to unsaturated cis- or trans-fatty acids (Figure 4C). Exendin-4 was unable to enhance these levels in cells treated with oleic acid. Providing exendin-4 after both elaidic and palmitic acids enhanced Beclin expression. LC3B gene expression also showed a similar trend except for oleic acid treatment. Here the expression levels were significantly suppressed by exendin treatment, although they still remained significantly higher than control (Fig. 4C).

**Figure 5:**
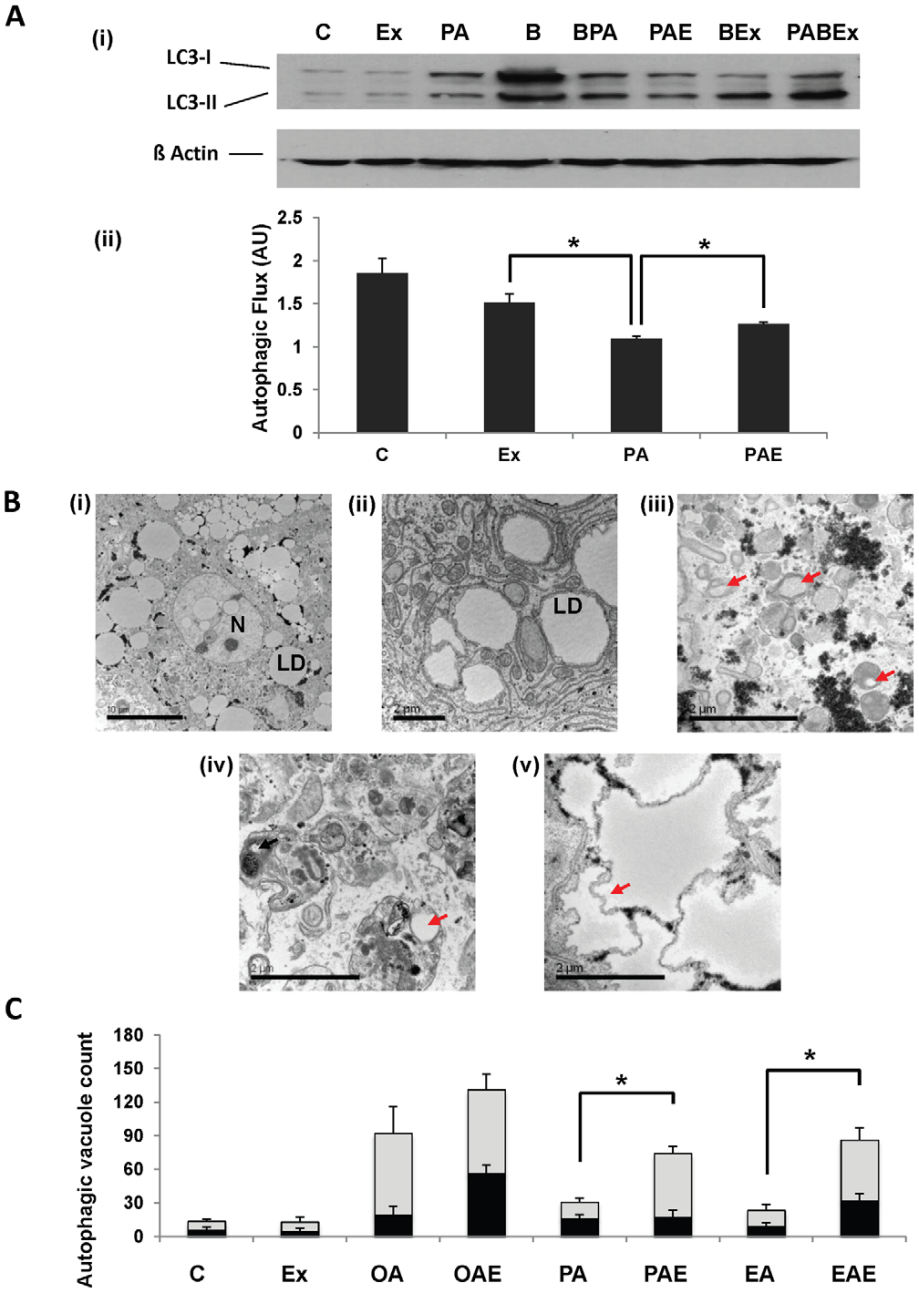
Exendin-4 stimulates autophagic vacuoles formation in human hepatocytes. (**A**) (i) Immunoblot for LC3 in palmitic acid and/or exendin-4 treated samples in presence or absence of bafilomycin A1. (***B***: bafilomycin A1; ***BPA***: bafilomycin with palmitic acid; ***BEx***: bafilomycin with exendin-4; ***PABEx***: palmitic acid with bafilomycin and exendin-4). (ii) Autophagic flux depicted as values obtained after taking the ratio of densitometry values of LC3-II values from bafilomycin treated samples to their respective bafilomycin-free counterparts. Note the increase in autophagic flux in response to exendin-4 in the presence of palmitic acid against palmitic acid alone. (**B**) Electron microscopy of primary human hepatocytes (i) showing fat loading, (***N***: nucleus; ***LD***: lipid droplet), (ii) structure of lipid droplets, (iii) autophagosomes containing lipid droplets (red arrow), (iv) autophagolysosome with lipid autophagosome and lipid droplet (iv) and, (v) shriveled lipid droplet (red arrow). Magnification: (i) – (ii) *x3.5E10^2^*, (iii) – (v) *x25E*10^3^. (**C**) Quantification of autophagic vacuoles (autophagosomes containing lipid droplets – dark bands, and autophagolysosomes containing lipid droplets – light bands) by integer scoring. #p<0.001, *p<0.05, Student'*s t-test,* three independent experiments were performed.

Another type of autophagy, chaperone mediated autophagy (CMA) has also been implicated in clearing toxic stress from cells. CMA is known to target proteins that carry a signal peptide and can occur in the absence of autophagosomes but presence of lysosomes. Lysosomal membrane associated protein LAMP2A has been identified as a marker for this mechanism. We investigated the profile of LAMP2A in exendin-4 treated and control fat-loaded hepatocytes. While there was a marked increase in the protein levels of LAMP2A in cells treated with plamitic acid followed by exendin-4 treatment, levels were relatively lower in hepatocytes treated with elaidic acid (Fig. 4A, B). Oleic acid and exendin-4 treatment on the other hand did not reveal a difference in the resulting amount of LAMP2A. LAMP2A gene expression on the other hand showed an increase in elaidic- and palmitic acid treatments, while it was suppressed in oleic acid after exendin-4 treatment (Fig. 4C). Additionally, to confirm if exendin was actually affecting the autophagic flux, cells were treated with bafilomycin (a vacuolar-type H+-ATPase inhibitor). Since bafilomycin inhibits the fusion of autophagosomes to lysosomes, we estimated the accumulation of autophagosomes by measuring LC3-II under bafilomycin treated and untreated conditions between cells treated with palmitic acid and/or exendin-4. Under bafilomycin treated conditions there was an increase in LC3-II labeling in contrast to non-bafilomycin conditions (Fig 5A (i)). To estimate autophagic flux, the ratio of densitometry values of LC3-II bands from control, palmitic acid, exendin and palmitic acid + exendin in presence of bafilomycin to their respective bafilomycin-free samples was taken. The result (Fig. 5A (ii)) shows that while palmitic acid suppresses autophagic flux, exendin-4 increases the flux even in the presence of palmitic acid. It should be noted that while exendin appears to be lowering the autophagic flux in comparison to control, the difference is not statistically significant.

To confirm if the increase in autophagy related genes actually resulted in an increased number of autophagosomes and whether there was indeed more lipophagy, we examined samples by transmission electron microscopy (TEM). Total number of autophagosomes that had lipid droplets in them and the total number of autophagolysosomes (AL) with lipid droplets were measured. Together these bodies are taken as autophagic vacuoles (AV) [Fig. 5B (i–iv)]. Exendin treatment increased the number of AVs, although the number of autophagosomes and ALs varied with treatment. In oleic acid treated hepatocytes there was an insignificant change in AVs after exendin treatment, although the autophagosome count was significantly increased by exendin-4. There was a clear increase in both autophagosomes and ALs under palmitic acid and exendin treatments (Fig. 5C). Elaidic acid treatment with or without exendin resulted in a similar number of autophagosomes, however, exedin-4 treatment significantly increased the number of ALs (Fig. 5C).

While visualizing cells for AVs we observed that some large sized lipid droplets had ‘shriveled’ margins with distinct absence of autophagic vacuoles around them [Fig. 5 (v)]. We hypothesized that this may be a result of change in contents of the lipid droplet, perhaps due to transport of fatty acids for beta oxidation. To confirm enhanced β-oxidation we determined the concentration of ketone bodies, the final breakdown product of beta oxidation. ß hydroxybutyrate served as a marker for oxidation. Exendin-4 treatment increased the production of ketone bodies in all the treatments in comparison to control. Fatty acids themselves also led to an increase in ketone bodies probably as a normal cellular response, which was further enhanced by exendin-4. The difference between exendin treated and untreated fat loaded cells was insignificant in the case of oleic acid. In contrast exendin treatment increased significantly ketone body formation in cells loaded with either palmitic or elaidic acid exposure (Fig. S3).

### American Lifestyle-Induced Obesity Syndrome (ALIOS) Model

**Figure 6:**
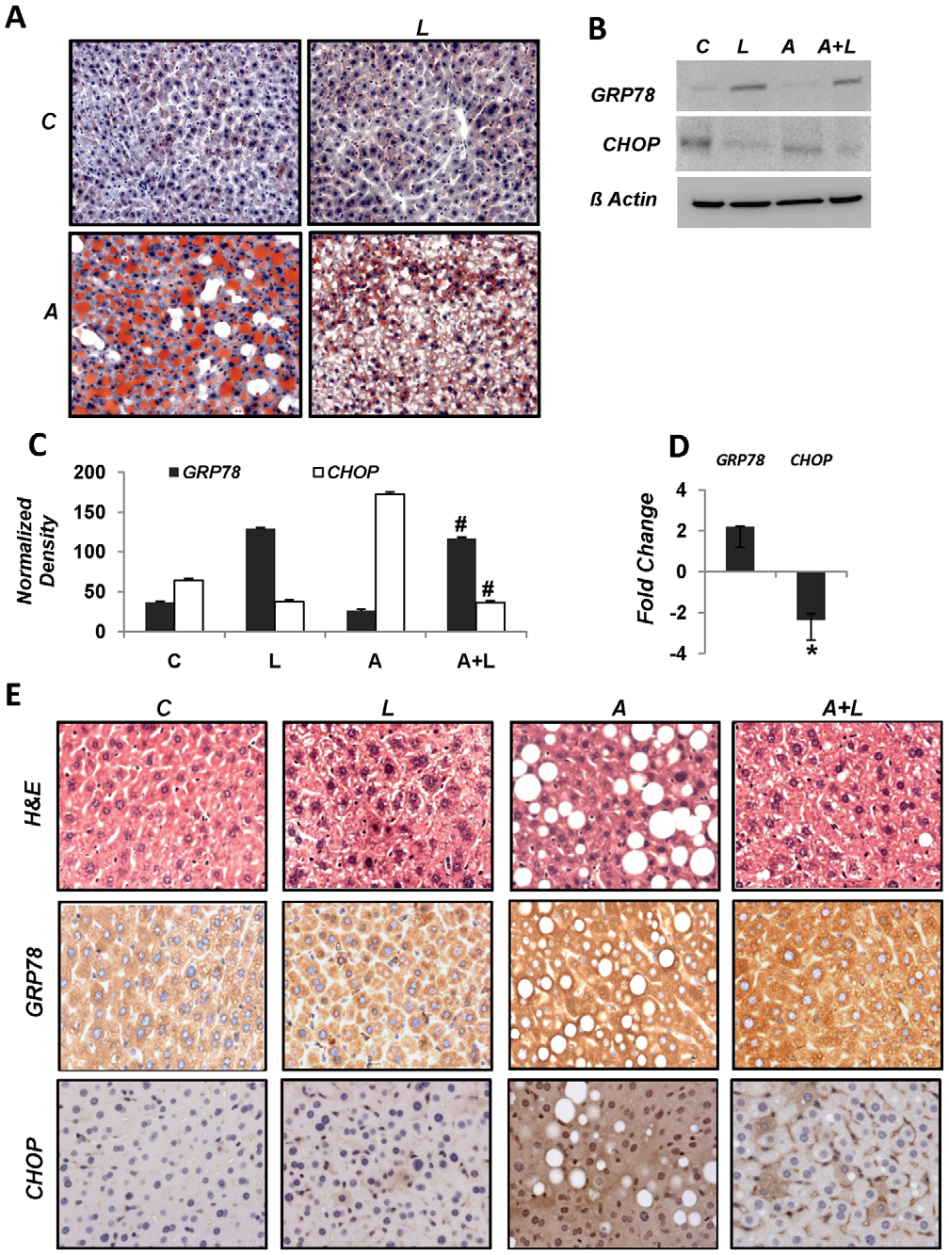
Liraglutide treatment reduces steatosis and ER stress in high fat diet fed mice. Mice were fed American Lifestyle-Induced Obesity Syndrome (ALIOS) diet and high fructose corn syrup for 8 weeks. One set of animals was subsequently treated with 200 µg/kg body weight liraglutide for next 4 weeks while maintaining other on high fat high sucrose diet. (**A**). Oil Red O staining of liver sections from mice kept on normal chow and treated with saline [*C*], liraglutide [*L*] and those given ALIOS diet alone [*A*] and subsequently liraglutide [*AL*]. (**B**) Immunoblotting of mouse liver lysates for GRP78 and CHOP. (**C**) Densitometric analysis of immunoblot (***C***: control, ***L***: liraglutide treated, ***A***: ALIOS fed, ***A+L***: ALIOS fed and then liraglutide injected). (**D**) Real time mRNA quantification of GRP78 and CHOP. (**E**) Immunohistochemical staining of mouse liver sections for GRP78 and CHOP. All studies were conducted at least three times in triplicate with fresh lysates, *p<0.05 Student'*s t-test.*

**Figure 7:**
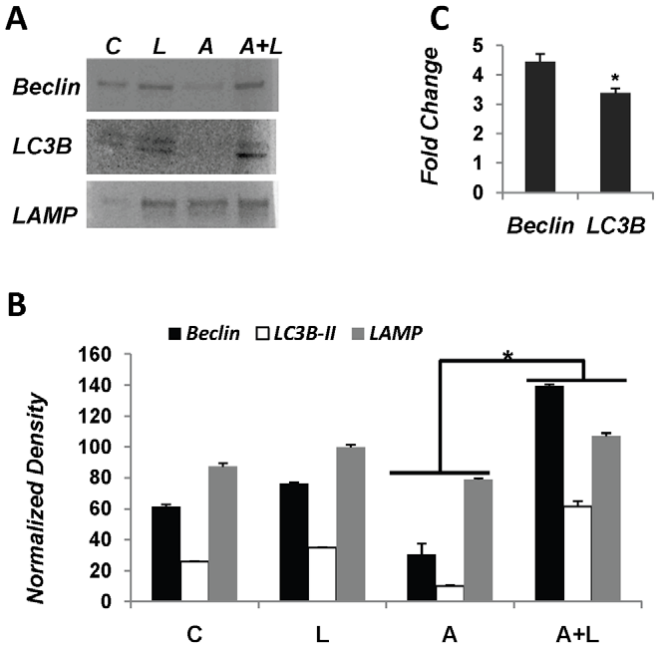
ALIOS-fed mice treated with liraglutide confirm GLP-1 enhanced lipoautophagy in liver. (**A**). Immunoblot comparing beclin, LC3B conversion and LAMP2 levels in liver lysates from mice treated with liraglutide or ALIOS diet or combination thereof. (**B**) Quantification of immunoblot by densitometry. Differences between protein levels were significant for respective genes between ALIOS and ALIOS+Liraglutide samples (**C**) Real time PCR analysis of Beclin, LC3B and LAMP2 gene expression. Histogram shows fold change in ALIOS-fed, liraglutide treated animals versus ALIOS fed animals. These experiments were performed with lysates from six mice, *p<0.05 Student'*s t-test*.

Oil red O staining of liver sections from animals fed normal chow, ALIOS diet and subsequently treated with liraglutide illustrated a marked reduction in the lipid load in hepatocytes. Also, there was a clear reduction in hepatic steatosis in drug treated animals (Fig. 6A). We further investigated the liver lysates for markers of UPR by immunoblotting and immunohistochemistry. Immunoblotting revealed suppression of GRP78 levels in animals fed ALIOS diet. Liraglutide injections in animals fed ALIOS diet reversed this effect by increasing GRP78 (Fig. 6B). Livers from animals administered the ALIOS diet demonstrated significant increases in CHOP protein in comparison to those given normal chow. In contrast, liraglutide treated mice had a remarkable reduction in CHOP protein levels (Fig. 7B). Densitometric analysis of the immunoblots confirmed these observations (Fig. 6C). In order to confirm if this difference in protein quantities was due to a transcriptional or post-transcriptional event, RT-qPCR analysis was carried out for these two genes. Results revealed that liraglutide treatment enhanced GRP78 and suppressed CHOP expression (Fig. 6D). We extended these studies by visualizing GRP78 and CHOP protein levels in liver sections by immunohistochemistry. Differences in the amount of signal for GRP78 and CHOP were similar to those observed by immunoblotting (Fig. 7A).

Investigations by Liu et al. (2009) have demonstrated that high fat diet leading to hyperinsulinemia and insulin resistance suppresses autophagy. In order confirm this in animals fed the ALIOS diet, we investigated autophagy markers beclin1, LC3B conversion, and LAMP2 in mouse livers. As is evident from Fig. 7B, beclin1 protein levels were suppressed in animals given ALIOS diet. Liraglutide injections in these animals increased beclin1 protein levels in liver. Similarly, LC3B-I was higher in animals fed high fat diet, whereas, LC3B-I was converted to LC3B-II in animals additionally injected with liraglutide. Examination of the lysosomal marker LAMP2 also considered a chaperone mediated autophagy marker revealed similar differences between high fat diet fed and GLP-1 analog treated mice. Densitometry of these immunoblots substantiated these findings (Fig. 7C). RT-qPCR analysis of beclin1 and LC3B disclosed that the increase in protein levels was an outcome of four-fold and three-fold increase in expression levels of beclin1 and LC3B genes, respectively (Fig. 7D). Taken together these findings confirm that while a Western diet (ALIOS) can suppress autophagy, GLP-1 analog treatment can reverse autophagic inhibition in mouse liver.

## Discussion

NAFLD refers to the spectrum of liver diseases in which steatosis can, in some humans, progress to more significant pathology including NASH and NASH-related cirrhosis. The accumulation of fat in hepatocytes is a consequence of three principle sources: *de novo* lipogenesis in the liver; nutritional uptake from the small intestine; and free fatty acid release from visceral white adipose tissue [19]. In one way or another each of these fatty acid reservoirs is a consequence of a diet high in sugar (e.g. high fructose corn syrup) or fatty acids that are highly saturated or have an all *trans-* structure. This diet results in both peripheral and hepatic insulin resistance. Recent data, however, indicate that the production of triglycerides may be a safety valve for hepatocytes prone to danger from lipotoxicity, the term used to describe the potential harm of long chain, saturated fatty acids. In this study we investigated whether the GLP-1 analogues could augment protective mechanisms in human hepatocytes that accumulated specific fatty acids; or in a mouse-feeding model with a diet as described above and consumed by Westerners.

Fatty acids available from the high fat diet are known to induce ER stress in hepatocytes [1]. Persistent ER stress results in rapid accumulation of unfolded proteins, which triggers apoptosis [20], [21], [22]. Gores recently demonstrated that free fatty acids loaded in mouse hepaotcytes directly engaged the apoptotic machinery by activating Bax, in a c-jun N-terminal kinase-dependent manner [1]. Both our *in vitro* and *in vivo* results here concur with their observations. As evident from Figure 2A, different fatty acids impart differential viability to hepatocytes, suggesting that all fatty acids are not equally toxic. On the other hand, earlier work on GLP-1 analogs has demonstrated protection of pancreatic ß-cells from ER stress and apoptosis [15], [23]. Since we recently identified the presence of GLP-1 receptors on the human hepatocyte [5], we set out to investigate potential mechanisms whereby GLP-1 reduces the free fatty acid burden in human cells. *In vitro* experiments presented here revealed that saturated, cis- and trans-unsaturated fatty acids have a variable effect on hepatocyte survival. Cell viability was reduced following fat loading and correlatively cell death was an outcome of apoptosis, as observed by an increase in cleaved caspase 3 and DAPI imaging. We also observed a marked reduction in cleaved caspase 3 following treatment with exendin-4 in cells containing fat when compared to non-fat loaded hepatocytes. We speculated that cell survival was the consequence of enhanced capacity to handle the unfolded protein response (UPR) and thereby prevent hepatocyte cell death via apoptosis.

GRP78 is the cardinal chaperone protein that binds to unfolded proteins and reduces ER stress, preventing ATF-6 activation that in turn promotes CHOP expression and initiates downstream apoptosis effectors [24]. We observed that exendin-4 induced the expression of GRP78 in hepatocytes and liraglutide did the same in mouse livers fed a high fat high fructose diet. These data suggest that while free fatty acids result in an increase in unfolded protein accumulation, there is an insufficient or effective level of GRP78 to abate the unfolded protein response. Exendin-4 on the other hand increased the amount of GRP78. Furthermore, if ER stress persists; C/EBP homologous protein (CHOP) activation can promote an imbalance between survival and apoptosis in favor of the latter. CHOP is ubiquitously expressed at very low levels. However, it is robustly expressed by perturbations that induce stress [25]. As we observed *in vitro*, FFA induced stress leads to an accumulation of CHOP. This observation is in contrast to that of Puri et al. [17], where CHOP expression was suppressed in NAFL and NASH in human liver samples. We demonstrated here that CHOP levels were increased in hepatocytes that showed high steatosis with either of the fatty acid types. This expression is reversed following exendin-4 treatment. These data were also seen following liraglutide therapy in the ALIOS-fed mice. Taken together, increased availability of the chaperone GRP78 and reduction in CHOP expression provides stress-relief to hepatocytes, suppressing apoptosis and promoting hepatocyte survival.

We are currently investigating the specificity of this effect of GLP-1 via upstream mechanisms related to the G-protein coupled receptor (GPCR). GLP-1R since it has been shown previously that GLP-1 may act through cAMP and exhibit its protective effects through the activation of protein kinase B/Akt through a cAMP-dependent phosphorylation of cAMP-responsive element-binding protein (CREBP) [26], [27], [28]. Immunohistochemical staining of liver sections for GRP78 and CHOP in mice given ALIOS diet and liraglutide suggest that GLP-1 analogs impart a strong affect in liver; though reduction in hepatic steatosis could as well be a result of whole body response to liraglutide, since GLP-1 receptors are present in other organs also. Recently there has been a surge of studies on the role of autophagy in maintaining cell signaling and health particularly in relation to diseases [29], [30]. Li et al. [31] have demonstrated that in mammalian cells knockdown of GRP78 leads to a reduction in autophagosome formation, though the conversion of LC3-I to LC3-II was not affected. Autophagy has also been implicated in cell death via apoptosis. Depending upon the kind and severity of stress it is possible that autophagy may determine cell fate [32], [33]. Most of the studies investigating autophagy have used starvation as a source of its induction. Physiologically autophagy occurs not only in response to starvation but also as a homeostatic process, conserved in all eukaryotes, whereby cellular contents can be delivered to the lysosome to yield recyclable nutrient components and rid cells of potentially deleterious proteins, organelles and pathogens [29]. Only recently studies by Singh et al. [10] have demonstrated autophagy to be targeting lipids inside the cell — and the concept of lipoautophagy emerged. Although we had no prior information suggesting that GLP-1 proteins promote autophagy, the disappearance of fatty acids following *in vitro* exendin-4 treatment prompted us to examine this process.

Exendin-4 increased the rate of autophagosome and autophagolysosome formation or the autophagic flux. Furthermore, exendin-4 induced key protein makers of autophagy both at the mRNA and protein levels. These data were identical in both the *in vitro* and *in vivo* models employed here. Beclin and LC3B were increased in mice treated with liraglutide. High fat diet suppressed the expression of these genes and consequently their proteins. These observations are consistent with those made by Liu et al. [34] where they demonstrated that autophagy is suppressed when mice are fed a high fat diet. Additionally, we observed an increase in LAMP2A. This protein is associated with the lysosomal membrane and is a key component of chaperone mediated autophagy (CMA). While we did not investigate CMA mechanisms it is possible that CMA is also activated in response to exendin-4 along with macroautophagy. LAMP2A has been shown to be an important component of lysosomes involved in fusion of the autophagosomes to lysosomes [35]. Investigations by Koga et al. [36] have revealed that mice fed a high fat diet had an autophagosome/lysosome fusion disorder. In our studies the high fat diet also suppressed markers of autophagy. These markers, however, were substantially increased transcriptionally in GLP-1 analog treated mice. The mechanism by which GLP-1 analogs induce such an effect needs further investigation. We, therefore, assume that an increase in macroautophagy guides the cell to increase in the number of lysosomes to accommodate increased flux of autophagosomes.

In addition to the increased number of autophagic vacuoles, larger lipid droplets showed ‘shriveled’ edges in absence of any autophagic vacuole. This prompted us to launch an investigation if there markers of enhanced ß-oxidation in response to exendin-4 could be detected. We examined culture media for the release keto-bodies (ß-hydroxybutyrate) and found an increase in these bodies in samples treated with exendin-4 (Fig. S3). Studies have reported that GLP-1 does not lead to an increase in ß-oxidation in mice pancreatic cells [37], [38]. Our data alone cannot implicate that GLP-1 directly increases ß-oxidation. It is tempting to speculate, however, that oxidation may be a result of decreased stress and fat load via autophagy, which is enhanced at the transcriptional level by exendin-4 and is further maintained by an increase in GRP78; hence additional experiments will be required to gain insight into the exact mechanisms of autophagic regulation by GLP-1 analogs. Down-regulation of the insulin receptor (IR) protein levels in insulin-target tissues such as liver, skeletal muscle, and adipose tissue has been shown to correlate with insulin resistance [39], [40], [41]. Zhou et al. [42] have identified an interesting link between ER stress, autophagy, and insulin resistance. They have demonstrated that obesity induces insulin resistance by ER stress-dependent down-regulation of the IR; and, ER stress-stimulated IR degradation is mediated by the autophagy-dependent process. They also demonstrated that the expression levels of IR were negatively associated with CHOP in insulin target tissues of *db*/*db* mice and mice fed a high-fat diet. In the work presented here we provide a plausible series of mechanisms associated with GLP-1 analog treatment that result in a beneficial relationship between autophagy and ER stress in hepatic steatosis. Arguably, GLP-1 in our work reduced ER stress and promoted autophagy, which, in turn, lead to reduction in both fat load and unfolded proteins. The results of our studies both *in vitro* and *in vivo* reveal restoration of normal metabolism in hepatocytes and consequently improve their chances for survival. Taken together, we have demonstrated that GLP-1 agonists can rescue hepatocytes from toxic fatty acids *in vitro*, or steatosis *in vivo,* by promoting autophagy and mitigating ER-stress mediated apoptosis. Further work to consider GLP-1 agonists for the treatment of human NAFLD is warranted both to fully understand the pathogenesis of disease and potentially to prevent disease progression.

## Materials and Methods

### Primary hepatocyte culture fatty acid loading and staining

Primary human hepatocytes were obtained through the Liver Tissue Cell Distribution System, (University of Pittsburgh, PA), funded by NIH Contract # N01-DK-7-0004/HHSN26700700004C. Cultures were maintained in BEBM^®^ medium (Lonza, Allendale, NJ) with BEGM^®^ SingleQuots (Lonza). For all *in vitro* experimental procedures hepaotcytes were grown and maintained in the absence of insulin. Protein and RNA were subsequently extracted. Long-chain fatty acids were purchased from Sigma Aldrich (St. Louis, MO), palmitic acid (16∶0), oleic acid (18∶1 cis), and elaidic acid (18∶1 trans). Untreated cells at 48 h did not have significantly different survivability rates when compared to cell survival at plating upon arrival.

All three fatty acids were dissolved in isopropanol to obtain a concentration of 100 mM. The required volume of the fatty acid stock was added to the medium containing 1% bovine serum albumin to obtain an 800 µM concentration of fatty acids in experiments. This concentration of fatty acids was similar fasting plasma concentrations of fatty acids under human nonalcoholic steatohepatitis conditions [43], [44]. The concentration of the vehicle, isopropanol, was less than 1% in final culture media. Cells were treated with each fatty acid independently and in combination of all the three at 200 µM each. Cells that were treated with the mix of all three fatty acid were unable to survive after 24 hours (data not shown). Cells were treated with fatty acid containing media for 24 h after which the media were changed and supplemented with 10 nM exendin-4 (Sigma) while maintaining fatty acid stress. Hepatocytes that were not provided exendin-4 were cultured for an additional 24 h. After treatment with exendin-4 for 24 h cells were harvested for staining with Oil Red O.

ORO stain (Sigma) was used to stain the cells after fixing as per following protocol. The cells were then visualized under the Olympus IX-51 (Center Valley, PA) inverted microscope at various magnification. To quantify the change in Oil Red O staining between treatments, protocol published by Dragunow et al. [45] was employed. The extracts were observed under the Synergy 2 spectrophotometer at 510 nm wavelength using Gen5 software (Bio-Tek, Winooski, VT).

### Cell survival assay

Cultured hepatocyte-viability following treatments with fatty acids and/or exendin-4, was evaluated using the Cell Proliferation Kit II (Roche, Indianapolis, IN) as per the manufacturer's protocol. Briefly, cultured cells were rinsed and treated with XTT for 12 h before reading absorbance of the modified dye at 680 and 492 nm. Assays were performed in triplicates. DAPI staining was resorted to visualize DNA condensation as a response to apoptosis.

### Preparation of cell lysates

Cells were harvested by centrifugation, washed with PBS, and resuspended in 0.1mL of lysis buffer containing 1% Triton X-100, 10mM Tris, pH 7.6, 50mM NaCl, 0.1% BSA, 1mM phenylmethyl sulfonyl fluoride (PMSF), 1% aprotinin, 5mM EDTA, 50mM NaF, 0.1% 2-mercaptoethanol, 5mM phenylarsine oxide, and 100mM sodium orthovanadate. Cell lysates were pre-cleared by centrifugation at 14,000×*g* at 4°C for 20min.

### RNA analysis

Total RNA was extracted from liver tissue and hepatocyte cell culture using the Qiagen RNeasy Mini Kit (Qiagen, Valencia, CA). Equal amounts of total RNA was used to synthesize the first DNA strand by iScript cDNA Synthesis Kit (Bio-Rad, Hercules, CA). Real-time quantitative PCR (RT-qPCR) was performed with the Eppendorf Realplex4 (Hauppauge, NY) thermocycler, using SYBR green (BioRad, Hercules, CA). Primer sequences used are listed in Table 1. All samples were run in triplicate and PCR products were normalized to 18s rRNA expression levels. Additionally, actin expression levels were also determined to confirm any changes in 18s levels between treatments (Fig. S2).

**Table 1:** Primers used for real-time PCR.

Organism	Gene	Primer
**Human**	GRP78 Forward	GCCGTCCTATGTCGCCTTC
	GRP78 Reverse	TGGCGTCAAAGACCGTGTTC
	CHOP Forward	TGGAAGCCTGGTATGAGGAC
	CHOP Reverse	TGTGACCTCTGCTGGTTCTG
	BECN1 Forward	ACCTCAGCCGAAGACTGAAG
	BECN1 Reverse	AACAGCGTTTGTAGTTCTGACA
	LC3B Forward	AAGGCGCTTACAGCTCAATG
	LC3B Reverse	CTGGGAGGCATAGACCATGT
	LAMP2 Forward	TTGGTTAATGGCTCCGTTTTCA
	LAMP2 Reverse	ACAAGGAAGTTGTCGTCATCTG
	XBP1 Forward	TTACGAGAGAAAACTCATGGCC
	XBP1 Reverse	GGGTCCAAGTTGTCCAGAATGC
**Mouse**	GRP78 Forward	CTGAGGCGTATTTGGGAAAGAA
	GRP78 Reverse	TGACATTCAGTCCAGCAATAGTG
	CHOP Forward	CTGGAAGCCTGGTATGAGGAT
	CHOP Reverse	CAGGGTCAAGAGTAGTGAAGGT
	BECN1 Forward	ATGGAGGGGTCTAAGGCGTC
	BECN1 Reverse	TCCTCTCCTGAGTTAGCCTCT
	LC3B Forward	TTATAGAGCGATACAAGGGGGAG
	LC3B Reverse	CGCCGTCTGATTATCTTGATGAG

### Immunoblotting and immunohistochemistry

Equal amounts of protein from cell and tissue lysates were resolved on SDS-PAGE [46] and transblotted on PVDF membrane (BioRad, Hercules, CA). A standard immunodetection protocol was employed using primary antibodies for GRP78 (Santa Cruz, location), CHOP, cleaved caspase 3, beclin, LC3B, LAMP2A (Cell Signaling) and ß-actin (Sigma) [47], and bafilomycin (Sigma). Samples were detected using HyGlo Chemiluminescent HRP Antibody Detection Reagent (Denville Scientific, Metuchen, NJ) on an automated Biospectrum Imaging System (UVP, Cambridge, UK).

### Assessment of autophagic flux

To assess the autophagic flux, cells were treated with palmitic acid as above, followed by 2.5 nM bafilomycin A1 (Sigma) treatment for 4 hrs. Subsequently the cells were treated with 10 nM exendin-4 in presence of palmitic acid and bafilomycin for an additional 6 hrs. After the end point cells were harvested for immunoblotting. Respective controls were maintained in which bafilomycin, palmitic acid and/or exnedin-4 were not added. Blotted membranes were subject to immunodetection with LC3 antibody followed by densitometry of the developed blots on ChemiDoc XRS+ using ImageLab software (BioRad, Hercules, CA, USA). To assess the autophagic flux between control, palmitic acid and/or exendin-4, ratio of the densitometry values of LC3-II bands of bafilomycin treated samples to respective bafilomycin-free samples was taken. The results thus obtained were plotted on a histogram to determine the change in the flux.

### Quantification of autophagic vacuoles by transmission electron microscopy (TEM)

Immediately after cell culture treatments were complete media was aspirated and 2.5% glutaraldehyde fixative [10 ml 25% glutaraldehyde (EMS, Fort Washington, PA), 50 ml 0.2 M cacodylate buffer, pH 7.4, 40 ml distilled water] was added to wells. Cells were left in fixative for a minimum of 2 hours at 4°C. Cells were then washed twice with 0.1 M cacodylate buffer at room temperature with agitation for 5 min. Following washes, 1% osmium fixative [2 parts 0.2 M cacodylate buffer, 1 part 6% potassium ferrocyanide (Sigma), 1 part 4% osmium tetroxide (EMS)] was added to cells and cells were left at room temperature for 1 h. Cells were then washed twice with 0.1 M cacodylate buffer, pH 7.4, at room temperature with agitation for 5 min. Fixed cells were dehydrated with a series of ethanol treatments (25%, 2 min; 50%, 2 min; 70%, 2 min; 95%, 2 min; 100%, 3×2 min). Following dehydration, cells were infiltrated with Epson resin (100% EtOH and Epson resin, 1∶1 for 1 hours followed by pure Epson resin, overnight).

After sections were cut and loaded on grid, they were observed under a Hitachi H-7500 Transmission Electron Microscope fitted with a Gatan BioScan 1K CCD camera, under various magnifications. Autophagic vacuoles (including autophagosomes and autophagolysosomes) were counted following the defined protocols [48], [49]. Briefly, the grids were loaded on the microscope and scanned systematically starting from the left edge at the bottom of the grid with surveillance of the specimen to score for AVs and ALs ascertaining that systematic sampling is achieved. Separate records of autophagosomes and autophagolysosomes were made to assess the total autophagic response.

### Mouse feeding studies

Male C57BL/J6 4-5 wk old mice were obtained from Jackson Laboratories (Bar Harbor, ME). All animal work was performed in accordance to relevant national and international guidelines. The work was approved by Emory University's Institutional Animal Care and Use Committee protocol # 065-2009. Animals were housed in laboratory cages at 23°C under a 12-hour light/dark cycle. Mice were fed either standard chow or the American Lifestyle-Induced Obesity Syndrome (ALIOS) diet as described by Tetri *et al.* [50]. The ALIOS diet provides 45% of calories from fat, with 30% of the fat in the form of partially hydrogenated vegetable oil [28% saturated, 57% monounsaturated fatty acids (MUFA), 13% polyunsaturated fatty acids (PUFA); trans-fat custom diet TD06303, Harlan Teklad)]. ALIOS-fed mice were also given high fructose corn syrup equivalents in their drinking water at 42 g/L (55% fructose, 45% glucose by weight) hereafter termed ALIOS-fed. Control mice were fed standard rodent chow. Food and water consumption were measured by weighing new and remaining food and volumes of water three times weekly. After 8 weeks on the diet, mice were injected with saline or 200 µg/kg of body weight liraglutide (Novo Nordisk, Princeton, NJ), daily for 4 weeks. Pair-feeding analysis and water consumption was not significantly different among different mouse cohorts including those ALIOS-fed mice compared to mice fed standard chow and water (data not shown).

### Statistical Analysis

All the experiments were conducted at least three times in triplicate. The data are presented as means ± standard error (SEM). Statistical analysis was performed using JMP v.8.01 (SAS Institute, Cary N.C.). Means were compared using the Student's *t*-test. The Tukey-Kramer test was also used to determine if any group differed significantly from each other.

## Supporting Information

Figure S1 **(A) ER stress establishment.** PERK phosphorylation was confirmed by immunoblot using phospho-PERK antibody (Santa Cruz # sc-32577). PERK phosphorylation was greater in fat loaded cells when compared to controls. Exendin-4 treatment of fatty acid loaded cells did not show significant difference from fatty acid loaded cells in the absence of exendin-4 treatment. **(B)**. **PCR for assessing XBP splicing.** XBP splicing was detected following the protocol of Cawley et al [1]. **(C)** XBP splicing in fatty acid loaded cells confirmed that ER stress was increased in hepatocytes; however, was reduced after exendin-4 treatment. We also observed splicing in exendin-4 treated samples in cells not loaded with fatty acids, though other markers of ER stress were not observed (c.f. Fig. 3). 1. Cawley K, Deegan S, Samali A, Gupta S (2011) Assays for detecting the unfolded protein response. Methods Enzymol 490: 31–51.
(TIF)

Figure S2 **Confirmation of lack of changes in 18s rRNA between treatments.** RT-qPCR of all samples for ß-Actin revealed absence of difference between expression of housekeeping gene 18s rRNA regardless of treatments.
(TIF)

Figure S3 **Exendin-4 induces fatty acid ß-oxidation.** Histogram showing increased levels of beta hydroxybutyrate in culture media, which was significantly increased (#: p<0.001, *: p<0.05) in media obtained from both palmitic and elaidic acid-loaded hepatocytes treated with exendin-4 as compared to oleic acid containing hepatocytes. The results are from three independent experiments, *p<0.05, Student'*s t-test*.
(TIF)

## References

[1] Malhi H, Gores GJ (2008). Molecular mechanisms of lipotoxicity in nonalcoholic fatty liver disease.. Semin Liver Dis.

[2] Centis E, Marzocchi R, Di Domizio S, Ciaravella MF, Marchesini G (2010). The effect of lifestyle changes in non-alcoholic fatty liver disease.. Dig Dis.

[3] Rask E, Olsson T, Soderberg S, Johnson O, Seckl J (2001). Impaired incretin response after a mixed meal is associated with insulin resistance in nondiabetic men.. Diabetes Care.

[4] Holst JJ (2004). On the physiology of GIP and GLP-1.. Horm Metab Res.

[5] Gupta NA, Mells J, Dunham RM, Grakoui A, Handy J (2010). Glucagon-like peptide-1 receptor is present on human hepatocytes and has a direct role in decreasing hepatic steatosis in vitro by modulating elements of the insulin signaling pathway.. Hepatology.

[6] Cazanave SC, Elmi NA, Akazawa Y, Bronk SF, Mott JL (2010). CHOP and AP-1 cooperatively mediate PUMA expression during lipoapoptosis.. Am J Physiol Gastrointest Liver Physiol.

[7] Wei Y, Wang D, Topczewski F, Pagliassotti MJ (2006). Saturated fatty acids induce endoplasmic reticulum stress and apoptosis independently of ceramide in liver cells.. Am J Physiol Endocrinol Metab.

[8] Ciechanover A (2005). Proteolysis: from the lysosome to ubiquitin and the proteasome.. Nat Rev Mol Cell Biol.

[9] Goldberg AL (2003). Protein degradation and protection against misfolded or damaged proteins.. Nature.

[10] Singh R, Kaushik S, Wang Y, Xiang Y, Novak I (2009). Autophagy regulates lipid metabolism.. Nature.

[11] Cuervo AM, Bergamini E, Brunk UT, Droge W, Ffrench M (2005). Autophagy and aging: the importance of maintaining “clean” cells.. Autophagy.

[12] Bandyopadhyay U, Sridhar S, Kaushik S, Kiffin R, Cuervo AM (2010). Identification of regulators of chaperone-mediated autophagy.. Mol Cell.

[13] Cunha DA, Ladriere L, Ortis F, Igoillo-Esteve M, Gurzov EN (2009). Glucagon-like peptide-1 agonists protect pancreatic beta-cells from lipotoxic endoplasmic reticulum stress through upregulation of BiP and JunB.. Diabetes.

[14] Yusta B, Baggio LL, Estall JL, Koehler JA, Holland DP (2006). GLP-1 receptor activation improves beta cell function and survival following induction of endoplasmic reticulum stress.. Cell Metab.

[15] Tsunekawa S, Yamamoto N, Tsukamoto K, Itoh Y, Kaneko Y (2007). Protection of pancreatic beta-cells by exendin-4 may involve the reduction of endoplasmic reticulum stress; in vivo and in vitro studies.. J Endocrinol.

[16] Bachar E, Ariav Y, Ketzinel-Gilad M, Cerasi E, Kaiser N (2009). Glucose amplifies fatty acid-induced endoplasmic reticulum stress in pancreatic beta-cells via activation of mTORC1.. PLoS One.

[17] Puri P, Mirshahi F, Cheung O, Natarajan R, Maher JW (2008). Activation and dysregulation of the unfolded protein response in nonalcoholic fatty liver disease.. Gastroenterology.

[18] Oyadomari S, Mori M (2004). Roles of CHOP/GADD153 in endoplasmic reticulum stress.. Cell Death Differ.

[19] Cusi K (2009). Role of insulin resistance and lipotoxicity in non-alcoholic steatohepatitis.. Clin Liver Dis.

[20] Wei Y, Wang D, Pagliassotti MJ (2007). Saturated fatty acid-mediated endoplasmic reticulum stress and apoptosis are augmented by trans-10, cis-12-conjugated linoleic acid in liver cells.. Mol Cell Biochem.

[21] Kim DS, Jeong SK, Kim HR, Chae SW, Chae HJ (2010). Metformin regulates palmitate-induced apoptosis and ER stress response in HepG2 liver cells.. Immunopharmacol Immunotoxicol.

[22] Pfaffenbach KT, Gentile CL, Nivala AM, Wang D, Wei Y (2010). Linking endoplasmic reticulum stress to cell death in hepatocytes: roles of C/EBP homologous protein and chemical chaperones in palmitate-mediated cell death.. Am J Physiol Endocrinol Metab.

[23] Bregenholt S, Moldrup A, Blume N, Karlsen AE, Nissen Friedrichsen B (2005). The long-acting glucagon-like peptide-1 analogue, liraglutide, inhibits beta-cell apoptosis in vitro.. Biochem Biophys Res Commun.

[24] Banhegyi G, Baumeister P, Benedetti A, Dong D, Fu Y (2007). Endoplasmic reticulum stress.. Ann N Y Acad Sci.

[25] Ron D, Habener JF (1992). CHOP, a novel developmentally regulated nuclear protein that dimerizes with transcription factors C/EBP and LAP and functions as a dominant-negative inhibitor of gene transcription.. Genes Dev.

[26] Gault VA, O'Harte FPM, Harriott P, Mooney MH, Green BD (2003). Effects of the novel (Pro3)GIP antagonist and exendin(9-39)amide on GIP- and GLP-1-induced cyclic AMP generation, insulin secretion and postprandial insulin release in obese diabetic (ob/ob) mice: evidence that GIP is the major physiological incretin.. Diabetologia.

[27] Hui H, Nourparvar A, Zhao X, Perfetti R (2003). Glucagon-like peptide-1 inhibits apoptosis of insulin-secreting cells via a cyclic 5′-adenosine monophosphate-dependent protein kinase A- and a phosphatidylinositol 3-kinase-dependent pathway.. Endocrinology.

[28] Jhala US, Canettieri G, Screaton RA, Kulkarni RN, Krajewski S (2003). cAMP promotes pancreatic beta-cell survival via CREB-mediated induction of IRS2.. Genes Dev.

[29] Huett A, Goel G, Xavier RJ (2010). A systems biology viewpoint on autophagy in health and disease.. Curr Opin Gastroenterol.

[30] Czaja MJ (2010). Autophagy in health and disease. 2. Regulation of lipid metabolism and storage by autophagy: pathophysiological implications.. Am J Physiol Cell Physiol.

[31] Li J, Ni M, Lee B, Barron E, Hinton DR (2008). The unfolded protein response regulator GRP78/BiP is required for endoplasmic reticulum integrity and stress-induced autophagy in mammalian cells.. Cell Death Differ.

[32] Ding W-X, Ni H-M, Gao W, Hou Y-F, Melan MA (2007). Differential effects of endoplasmic reticulum stress-induced autophagy on cell survival.. J Biol Chem.

[33] He C, Klionsky DJ (2009). Regulation mechanisms and signaling pathways of autophagy.. Annu Rev Genet.

[34] Liu HY, Han J, Cao SY, Hong T, Zhuo D (2009). Hepatic autophagy is suppressed in the presence of insulin resistance and hyperinsulinemia: inhibition of FoxO1-dependent expression of key autophagy genes by insulin.. J Biol Chem.

[35] Eskelinen EL, Schmidt CK, Neu S, Willenborg M, Fuertes G (2004). Disturbed cholesterol traffic but normal proteolytic function in LAMP-1/LAMP-2 double-deficient fibroblasts.. Mol Biol Cell.

[36] Koga H, Kaushik S, Cuervo AM (2010). Altered lipid content inhibits autophagic vesicular fusion.. FASEB J.

[37] Peyot M-L, Gray JP, Lamontagne J, Smith PJS, Holz GG (2009). Glucagon-like peptide-1 induced signaling and insulin secretion do not drive fuel and energy metabolism in primary rodent pancreatic beta-cells.. PLoS One.

[38] Sorhede Winzell M, Ahren B (2004). Glucagon-like peptide-1 and islet lipolysis.. Horm Metab Res.

[39] Garvey WT, Olefsky JM, Marshall S (1986). Insulin induces progressive insulin resistance in cultured rat adipocytes. Sequential effects at receptor and multiple postreceptor sites.. Diabetes.

[40] Boden G, Chen X, Ruiz J, Heifets M, Morris M (1994). Insulin receptor down-regulation and impaired antilipolytic action of insulin in diabetic patients after pancreas/kidney transplantation.. J Clin Endocrinol Metab.

[41] Venkatesan N, Davidson MB (1995). Insulin resistance in rats harboring growth hormone-secreting tumors: decreased receptor number but increased kinase activity in liver.. Metabolism.

[42] Zhou L, Zhang J, Fang Q, Liu M, Liu X (2009). Autophagy-mediated insulin receptor down-regulation contributes to endoplasmic reticulum stress-induced insulin resistance.. Mol Pharmacol.

[43] Belfort R, Harrison SA, Brown K, Darland C, Finch J (2006). A placebo-controlled trial of pioglitazone in subjects with nonalcoholic steatohepatitis.. N Engl J Med.

[44] Sanyal AJ, Campbell-Sargent C, Mirshahi F, Rizzo WB, Contos MJ (2001). Nonalcoholic steatohepatitis: association of insulin resistance and mitochondrial abnormalities.. Gastroenterology.

[45] Dragunow M, Cameron R, Narayan P, O'Carroll S (2007). Image-based high-throughput quantification of cellular fat accumulation.. J Biomol Screen.

[46] Handy JA, Saxena NK, Fu P, Lin S, Mells JE (2010). Adiponectin activation of AMPK disrupts leptin-mediated hepatic fibrosis via suppressors of cytokine signaling (SOCS-3).. J Cell Biochem.

[47] Sharma D, Wang J, Fu PP, Sharma S, Nagalingam A (2010). Adiponectin antagonizes the oncogenic actions of leptin in hepatocellular carcinogenesis.. Hepatology.

[48] Yla-Anttila P, Vihinen H, Jokitalo E, Eskelinen EL (2009). Monitoring autophagy by electron microscopy in Mammalian cells.. Methods Enzymol.

[49] Swanlund JM, Kregel KC, Oberley TD (2010). Investigating autophagy: quantitative morphometric analysis using electron microscopy.. Autophagy.

[50] Tetri LH, Basaranoglu M, Brunt EM, Yerian LM, Neuschwander-Tetri BA (2008). Severe NAFLD with hepatic necroinflammatory changes in mice fed trans fats and a high-fructose corn syrup equivalent.. Am J Physiol Gastrointest Liver Physiol.

