# Supplementary figures and images for: GLP-1 Analogs Reduce Hepatocyte Steatosis and Improve Survival by Enhancing the Unfolded Protein Response and Promoting Macroautophagy
Source: PLoS One. 2011 Sep 21;6(9):e25269. doi: 10.1371/journal.pone.0025269 (PMC3177901; doi:10.1371/journal.pone.0025269)

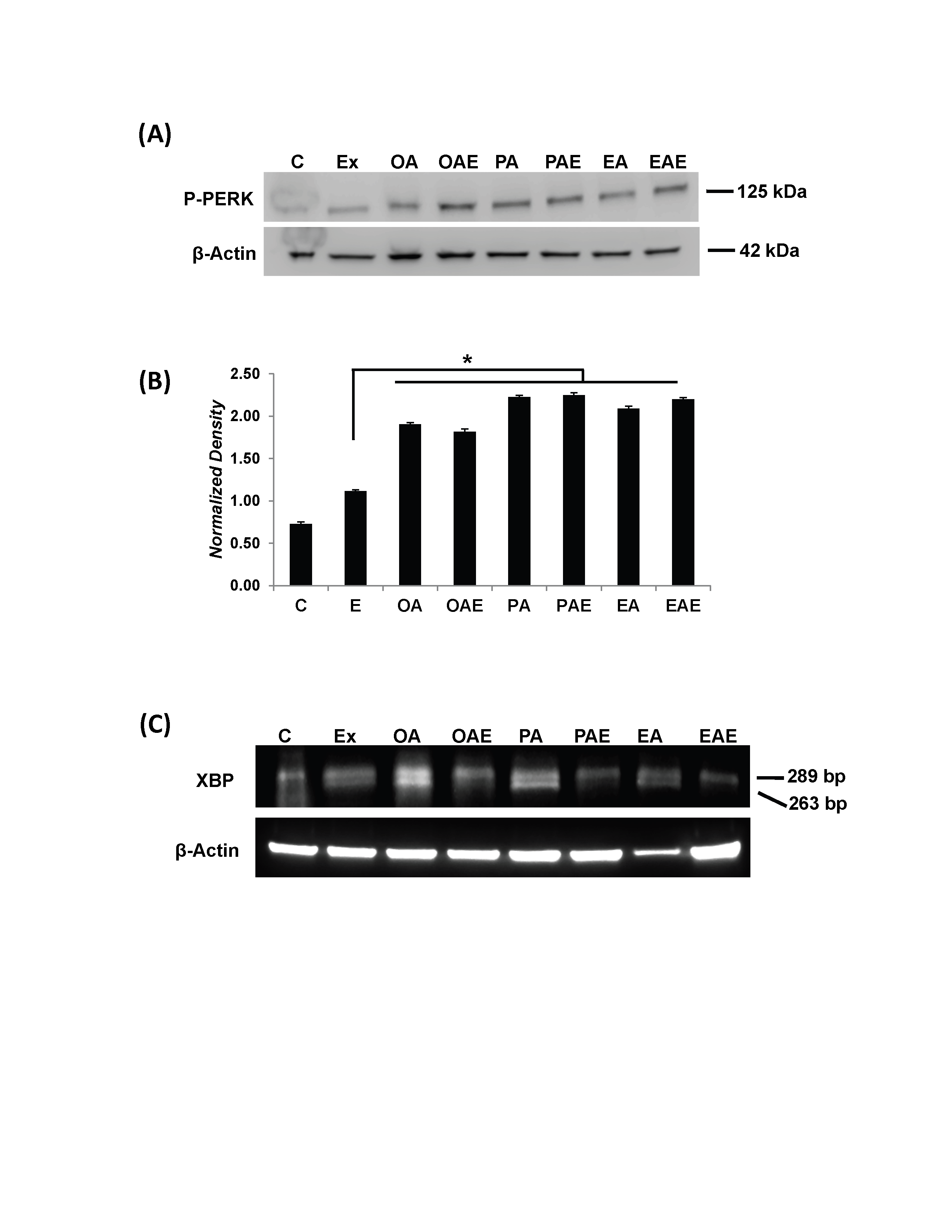

Supplement: Figure S1 — (A) ER stress establishment. PERK phosphorylation was confirmed by immunoblot using phospho-PERK antibody (Santa Cruz # sc-32577). PERK phosphorylation was greater in fat loaded cells when compared to controls. Exendin-4 treatment of fatty acid loaded cells did not show significant difference from fatty acid loaded cells in the absence of exendin-4 treatment. (B). PCR for assessing XBP splicing. XBP splicing was detected following the protocol of Cawley et al [1]. (C) XBP splicing in fatty acid loaded cells confirmed that ER stress was increased in hepatocytes; however, was reduced after exendin-4 treatment. We also observed splicing in exendin-4 treated samples in cells not loaded with fatty acids, though other markers of ER stress were not observed (c.f. Fig. 3). 1. Cawley K, Deegan S, Samali A, Gupta S (2011) Assays for detecting the unfolded protein response. Methods Enzymol 490: 31–51. (TIF) [file pone.0025269.s001.tif]

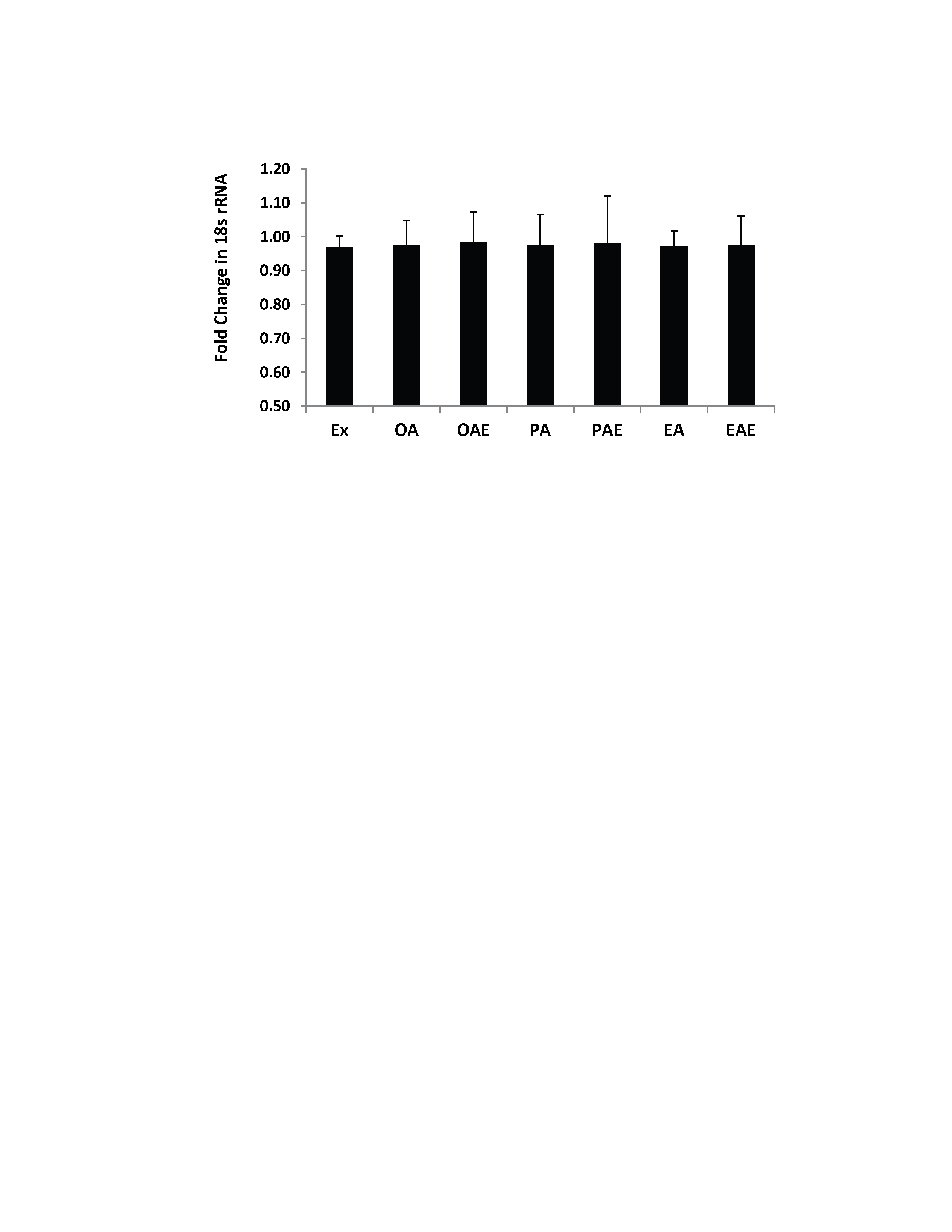

Supplement: Figure S2 — Confirmation of lack of changes in 18s rRNA between treatments. RT-qPCR of all samples for ß-Actin revealed absence of difference between expression of housekeeping gene 18s rRNA regardless of treatments. (TIF) [file pone.0025269.s002.tif]

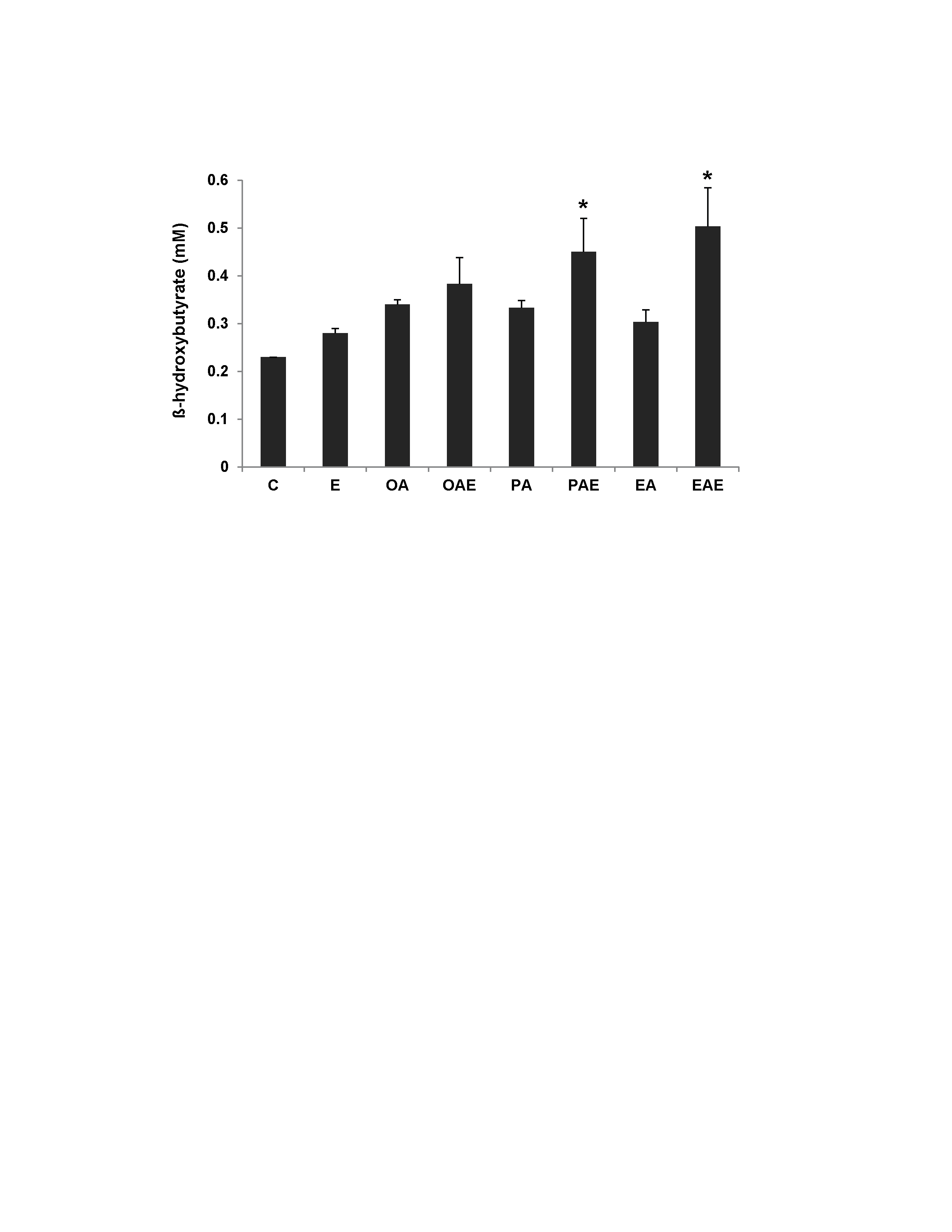

Supplement: Figure S3 — Exendin-4 induces fatty acid ß-oxidation. Histogram showing increased levels of beta hydroxybutyrate in culture media, which was significantly increased (#: p<0.001, *: p<0.05) in media obtained from both palmitic and elaidic acid-loaded hepatocytes treated with exendin-4 as compared to oleic acid containing hepatocytes. The results are from three independent experiments, *p<0.05, Student's t-test. (TIF) [file pone.0025269.s003.tif]
